# The association between the red cell distribution width and diabetic nephropathy in patients with type-2 diabetes mellitus

**DOI:** 10.1080/0886022X.2018.1532906

**Published:** 2018-10-29

**Authors:** Junlin Zhang, Rui Zhang, Yiting Wang, Hanyu Li, Qianqian Han, Yucheng Wu, Shanshan Wang, Ruikun Guo, Tingli Wang, Li Li, Fang Liu

**Affiliations:** Division of Nephrology, West China Hospital of Sichuan University, Chengdu, China

**Keywords:** red cell distribution width, diabetic nephropathy, type-2 diabetes mellitus, end-stage renal disease

## Abstract

**Background:** Red cell distribution width (RDW) has been reported to be involved in metabolic syndrome and cardiovascular events. Patients with diabetic nephropathy (DN) are often found to be with high level of RDW. The aim of this study was to explore whether RDW was associated with DN severity and progression in patients with type-2 diabetes mellitus (T2DM).

**Methods:** A total of 175 T2DM patients with biopsy-proven DN were enrolled. The baseline clinical and pathologic data of these patients was extracted from the medical records. The patients then were divided into two groups based on the median (13.6%) of RDW level; group 1: <13.6% and group 2: ≥13.6%. The effect of RDW level on the renal outcomes was evaluated by using cox regression analysis.

**Results:** Compared with the patients with lower RDW level, the patients with higher level of RDW had higher proportions of female, longer DM duration, lower levels of eGFR, albumin and hemoglobin, and more serious glomerular damage. Moreover, the RDW levels were negatively corrected with eGFR (*r* = −0.283, *p* < 0.001), but positively related with proteinuria (*r* = 0.227, *p* = 0.003). In the follow-up period, 81(46.3%) patients had reached ESRD from baseline. Importantly, the Cox regression analyses showed that the levels of RDM had a significant effect on the risk of progression to ESRD (HR = 1.92, *p* < 0.01), albeit not emerged as an independent predictor.

**Conclusions:** These data indicated that the levels of RDW were significantly associated with increased risk of progression to ESRD in patients with DN, despite did not an independent predictor.

## 1. Introduction

Diabetic nephropathy (DN) is one of the most common microvascular complications in patients with diabetes mellitus (DM) and mainly caused by continued exposure to high glucose condition [1]. In China, a national survey in 2015 showed that the prevalence of chronic kidney disease (CKD) related to diabetes has exceeded the percentage of CKD related to glomerulonephritis (1.23% vs. 0.91%), listed as the primary cause of CKD in the general population [2]. In the United States, the incidence of DN in the biopsy-proven kidney disease has also snowballed from 5.5% in the decade 1986–1995 to 19.1% in the decade 2006–2015 [3], ranked as the leading cause for end-stage renal disease (ESRD) [1,4]. Given its widespread incidence and huge socioeconomic toll [5], the prevention and management of DN and its complications are of great clinical and societal associations.

Previously, several studies have evidenced that proteinuria, eGFR, hemoglobin A1c (Hb1Ac), blood pressure and glomerular lesions are closely related with the progression of DN [6–8]. Red cell distribution width (RDW), as a marker of anisocytosis, is often used to differentiate the pathogenesis of anemia, but recently it has shown to be an effective risk factor for the morality of several diseases such as contrast-induced nephropathy [9], coronary heart disease (CHD) [10], stroke [11] and heart failure [12,13]. However, whether the RDW was associated with the DN severity and progression remains unknown.

In this study, we aimed to explore the relationship between the RDW level, baseline clinical and pathological features, and renal prognosis in 175 patients with T2DM and biopsy-proven DN.

## 2. Methods

### 2.1. Patients enrollment

**Figure 1.**
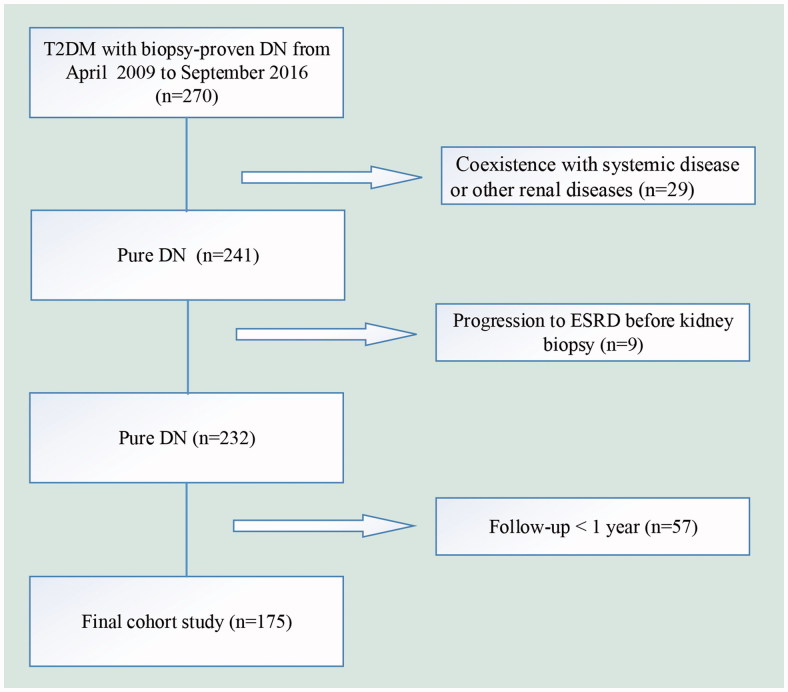
The flow chart for patients in this study. DN: diabetic nephropathy; ESRD: end-stage renal disease.

We enrolled 175 patients with T2DM and DN with a wide range of eGFR and proteinuria form 2009 to 2016 at the West China Hospital of Sichuan University. The study flowchart of participants exclusion was shown in Figure 1. The inclusion criteria in this study were (1) age ≥18 years old, (2) the diagnosis of DN proven by renal biopsy, (3) eGFR >15 ml/min/per 1.73 m^2^. The study was in compliance with the principles of Declaration of Helsinki and was approved by the Ethics Committee of West China Hospital of Sichuan University. Written informed consent was obtained from all participants.

### 2.2. Data collection and definitions

Clinical, laboratory and pathological data were extracted within 1 month of renal biopsy, including age, gender, weight, height, BP, 24-hour urinary protein, eGFR (calculated using the CKD-EPI equation), Hb1Ac, RDW and pathological classification. The renal end point of this study was the progression to ESRD from the baseline. ESRD was defined as requirement of treatment with chronic dialysis. Patients who did not reach endpoint were evaluated at their last follow-up visit. The classification of DN was based on the standards of the Renal Pathology Society (RPS) in 2010 [14].

### 2.3. Statistical analyses

We analyzed all data using SPSS22.0 software (SPSS, Chicago, IL). Continuous variables with normal distribution were presented as mean with standard deviation (SD); non-normal variables were reported as median with interquartile ranges (IQR, 25th and 75th percentiles). Variables were expressed as percentages for categorical variables. The differences of means among groups were compared by Student’s *t*-test or Mann–Whitney *U*-test, as appropriate. The frequencies of categorical variables were compared using Pearson χ^2^ or Fisher’s exact test, when appropriate. Analysis of renal survival was calculated with the Kaplan–Meier method. Univariate and multivariate Cox regression analysis evaluated the potential confounding factors of RDW in influencing renal progression. A two-sided *p* value <0.05 was considered as statistically significance.

## 3. Results

### 3.1. Baseline characteristics of the study

A total of 175 patients with T2DM and biopsy-proven DN were retrospectively evaluated (Figure 1). The clinical and laboratory information of the subjects was shown in Table 1. Median of RDW was 13.6 (13.1–14.4) % for the patients in this study. There were 94 (53.71%) patients with higher level of RDW (≥13.6%) and 81(46.29%) patients with lower level of RDW (<13.6%).

**Table 1.** Demographic and laboratory data of the patients according to RDW level.

Variable	All	RDW(%) < 13.6	RDW(%) ≥ 13.6	*p* value
	(*n* = 175)	(*n* = 81)	(*n* = 94)	
Age (years)	52 (47–59)	52 (48–56.5)	51.5 (47–60.25)	NS
Gender (Male, %)	122 (69.7)	68 (84.0)	54 (57.4)	<0.001
Duration of diabetes (Months)	84 (36–132)	72 (24–120)	108 (36–132)	0.007
DR (%)	85 (48.6)	38 (46.9)	47 (50.0)	NS
Cigarette smoking (%)	87 (49.7)	48 (59.3)	39 (41.5)	0.019
Body mass index (kg/m2)	25.37 (22.11–28.06)	24.73 (21.40–29.07)	25.55 (24.22–27.41)	NS
SBP (mm Hg)	146.98 ± 21.91	145.04 ± 21.25	148.66 ± 22.44	NS
DBP (mm Hg)	86.20 ± 12.49	86.52 ± 11.42	85.93 ± 13.40	NS
Hypertension (%)	151 (86.3)	66 (81.5)	85 (90.4)	NS
Hematuria (%)	86 (49.1)	35 (43.2)	51 (54.3)	NS
Initial proteinuria (g/d)	4.18 (1.83-7.01)	3.70 (1.55-5.88)	4.60 (2.22-8.66)	0.053
e-GFR (ml/min/1.73m2)	51.89 (37.96–78.71)	67.87 (44.00–90.92)	43.35 (32.53–76.07)	0.001
eGFR< 60 (ml/min/1.73m2,%)	98 (56.0)	33 (40.7)	65 (69.1)	<0.001
Serum creatinine (mg/dl)	1.39 (0.98–1.82)	1.22 (0.91–1.66)	1.56 (1.11–1.94)	0.013
Serum albumin (g/L)	33.73 ± 7.91	35.24 ± 7.01	32.42 ± 8.43	0.017
HbA1c (%)	7.05 (6.20–8.40)	7.50 (6.20–9.15)	6.80 (6.10–7.70)	0.056
Triglyceride (mmol/L)	1.79 (1.21–2.41)	1.83 (1.21–2.66)	1.74 (1.21–2.18)	NS
Total cholesterol (mmol/L)	5.08 (4.32–6.00)	5.16 (4.14–6.28)	5.00 (4.40–5.74)	NS
Hemoglobin (g/L)	117.99 ± 26.15	127.61 ± 24.62	109.70 ± 24.65	<0.001
RDW (%)	13.6 (13.1–14.4)	13.10 (12.75–13.25)	14.30 (13.80–15.03)	<0.001
Progression to ESRD	81 (46.3)	29 (35.8)	52 (55.3)	–
Therapy				
RAAS inhibitor (%)	141 (80.6)	69 (85.2)	72 (76.6)	NS
Oral hypoglycemic agents (%)	75 (42.9)	43 (53.1)	32 (34.0)	0.011
Insulin therapy (%)	127 (72.6)	51 (63.0)	76 (80.9)	0.008
Statins (%)	106 (60.6)	50 (61.7)	56 (59.6)	NS
Antiplatelet agents (%)	42 (24.0)	17 (21.0)	25 (26.6)	NS

DBP: diastolic blood pressure; DR: diabetic retinopathy; e-GFR: estimated glomerular filtration rate; ESRD: end-stage renal disease; HbA1c: glycosylated hemoglobin; NS: not significant; RAAS: Renin–angiotensin–aldosterone system; SBP: systolic blood pressure.

Data are presented as the mean ± standard, the median with IQR or counts with percentages. A two-tailed *p* < 0.05 was considered statistically significant.

### 3.2. Associations of RDW with baseline clinical parameters

The patients with higher level of RDW(≥13.6%) had higher proportions of female and insulin therapy, longer DM duration, lower levels of eGFR, albumin and hemoglobin, compared with those with lower RDW level (*p* < 0.05). However, the incidence of diabetic retinopathy, hypertension and hematuria, and the levels of BMI, HbA1c, triglyceride and total cholesterol were comparable between two groups (*p* > 0.05) (Table 1).

**Figure 2.**
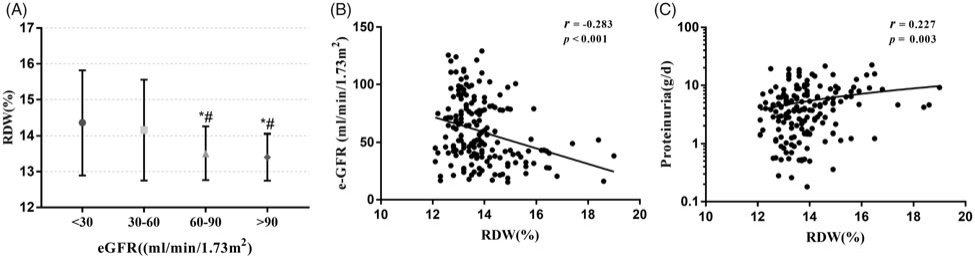
(A) Distributions of RDW by eGFR among 175 patients with DN. (B) Correlation of RDW with eGFR. (C) Correlation of RDW with urine protein. *p < 0.05 versus eGFR <30 (ml/min/1.73m2); #*p* < 0.05 versus eGFR between 30 and 60 ml/min/1.73 m^2^.

As shown in Figure 2(A), RDW levels of the patients with CKD stages 3 and 4 were higher than those with CKD stages 1–2. In addition, the RDW level was significantly negatively correlated with eGFR (*r* = −0.283, *p* < 0.001), whereas it was positively correlated with proteinuria (*r* = 0.227, *p* = 0.003) (Figure 2(B,C)). There were also significant correlations between RDW levels and DM duration (*r* = 0.203, *p* = 0.007), serum albumin (*r* = −0.243, *p* = 0.001), and hemoglobin (*r* = −0.386, *p* < 0.001).

### 3.3. Associations of RDW with baseline pathological parameters

Given that pathological changes could predict the renal prognosis of DN, we next assessed the relationship between the RDW level and pathological characteristics (Table 2). Compared the patients with lower level of RDW, the patients with higher RDW level had more serious glomerular lesions (*p* < 0.05). Whereas the interstitial fibrosis and tubular atrophy (IFTA), interstitial inflammation and arteriolar hyalinosis were comparable (*p* > 0.05) between the two groups. Moreover, the RDW level had a significant positive correlation with glomerular class (*r* = 0.227, *p* = 0.02).

**Table 2.** Pathological findings according to RDW levels.

Pathological lesions	All	RDW(%) < 13.6	RDW(%) ≥ 13.6	p* value
	(*n* = 175)	(*n* = 81)	(*n* = 94)	
Glomerular class				0.029
10	10	3	7	
II a	34	23	11	
II b	17	9	8	
III	86	37	49	
IV	28	9	19	
IFTA				0.338
0	6	2	4	
1	79	39	40	
2	74	37	37	
3	16	3	13	
Interstitial inflammation				0.474
0	12	5	7	
1	128	58	70	
2	35	18	17	
Arteriolar hyalinosis				0.274
0	25	11	14	
1	79	42	36	
2	71	28	43	

IFTA: interstitial fibrosis and tubular atrophy.

*Wilcoxon rank sum test.

### 3.4. RDW and ESRD

**Figure 3.**
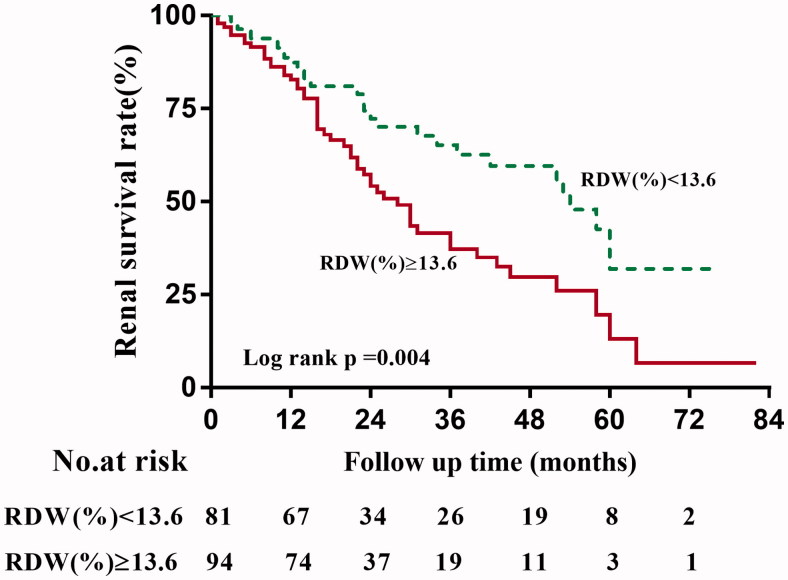
Kaplan–Meier curves of renal survival rates in patients with different levels of RDW.

During a median follow-up time of 18 (12–85) months, 81 of 175 (46.3%) patients developed ESRD. The Kaplan–Meier survival analysis suggested that the renal survival rates at first and third year were 87.29% and 65.11% for the group of RDW <13.6%, and 82.77% and 37.11% for the group of RDW ≥13.6% (*p* = 0.004), as shown in Figure 3.

**Figure 4.**
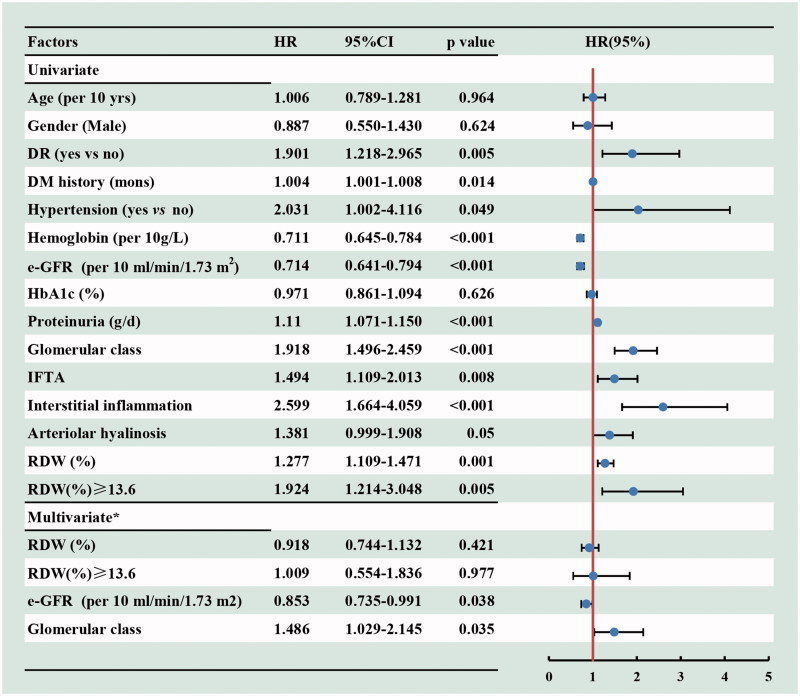
Cox regression analysis for incident ESRD according to baseline variables among 175 patients with DN. *Adjusted for baseline age, gender, DR (yes or no), hypertension (yes or no), DM duration, hemoglobin e-GFR, proteinuria and renal pathological findings (including the glomerular class, IFTA, interstitial inflammation score and arteriolar hyalinosis). 95% CI: 95% confidence interval; HR: hazard ratio; RDW: red cell distribution width.

The univariate Cox regression analyses indicated that the diabetic retinopathy, DM history, hypertension, eGFR, proteinuria, pathological changes and higher level of RDW (HR, 1.924, 95%CI, 1.214–3.048, *p* = 0.005) were associated with the progression of DN to ESRD. However, after adjusted for the baseline age, gender, DR, hypertension, DM duration, hemoglobin, e-GFR, proteinuria and renal pathological findings (including the glomerular class, IFTA, interstitial inflammation score and arteriolar hyalinosis), the higher level of RDW was not an independent risk factor for renal prognosis (HR, 1.009, 95%CI, 0.554–1.836, *p* = 0.977) (Figure 4).

## 4. Discussion

In the present investigation, we found that the higher RDW levels were significantly associated with declined eGFR, heavier proteinuria, lower levels of albumin and hemoglobin, and more severe glomerular damage, which represented the first examination of the relationship between the RDW and pathological changes. In addition, the results also indicated that the RDW level could influence the renal prognosis in patients with DN, albeit not being as an independent risk factor.

Several studies have reported that higher RDW level was correlated with proteinuria and decreased eGFR in patients with DM [15,16], which was consistent with our study. N Malandrino et al. [15] reported that the patients with higher RDW levels were more likely to be currently smoking and with hypertension. XF Xiong et al. [16] found that the DM duration and SBP level were positive associated with the RDW level, but the HbA1c was negative related with it. In this study, we confirmed that the RDW level was associated with smoking incidence and DM duration, but not with hypertension and HbA1c. Those discrepancies were most likely because some DM patients in their studies were without kidney disease, but the participants in this study were all with biopsy-proven DN.

Previous studies have evidenced that RDW could be as a risk factor for CVD morbidity and mortality in different populations [12,17–20]. And other studies have reported that the higher RDW was associated with renal function decline in unselected outpatients [21] and contrast-induced nephropathy incident in patients undergoing percutaneous coronary intervention for acute coronary syndrome [9]. However, RDW has not been reported as a predictor for incident ESRD in patients with DN. Although the results of several cross-sectional studies [15,16] observed that the RDW was associated with progression to DN in DM, weather it could further influence the renal prognosis remains unclear. In our study, although RDW level was not an independent risk factor for renal outcomes, this finding in no way reduced its potential influence on the prognosis of DN.

RDW is a widely available and routinely measured in the blood tests. Elevated RDW levels could be a consequence of various anemia. However, N Malandrino et al. [15] found that the association between RDW and DN development in patients with DM was independent of anemia, which suggested that other possible pathogenic mechanisms exist to exacerbate the disease progression. Inflammation could impact on erythropoiesis, shorten erythrocyte half-life and induce anisocytosis, resulting in an increased level of RDW [22]. Previously, a large body of studies have evidenced that the RDW level was significantly associated with CRP [23] and interleukin-6 (IL-6) [24], which were well-established inflammatory markers. DM and DN were also related with low grade inflammation [25,26], and increased level of CRP and IL-6 had been evidenced to be involved in the progression of diabetic complications [27]. Moreover, sufficient evidence has indicated that oxidative stress (OS) is an important factor in the development of DN [28], and has been linked with deranged hematopoiesis, ultimately causing anisocytosis [29]. That implied a possible effect of the underlying inflammatory state and oxidative stress, reflected as elevated RDW levels, on accelerated renal damage. However, the exact mechanism and signal pathways await further studies to determine.

It was unexpected that DR, as another common microvascular complication of DM, was not associated with the RDW level in our study, which was in line with the result of N Malandrino et al. [15]. Although several studies have shown that the inflammation was also probably involved in the pathogenesis of DR [27], other research suggested that DR was weakly or not at all associated with inflammatory markers, especially CRP [30–32]. Whether the RDW influence the development of DR remains controversial. Thus, more in depth basic and clinical research is necessary to further evaluate the association between RDW and DR.

The results of this study should be interpreted based on the study’s limitations. First, because of the retrospective design, we could not establish causal associations and related molecular mechanisms between the RDW and DN. Second, the measurement of RDW level was only detected once at baseline, the subsequent tests during the follow-up may help to further determine its association with the long-term prognosis of DN. Third, considering the CVD events were the outcomes with the most missing information, we did not further evaluate the relationship between RDW and CVD risk. Finally, we did not consider the therapeutic interventions, which might influence the renal prognosis as additional confounding factors.

In summary, this study demonstrated that higher RDW values were associated with increased risk of ESRD in 175 patients with DN, although this association was not independent. Beyond the classical clinical risk factors such as eGFR and albuminuria, RDW may be a potential biomarker to stratify high-risk patients before progression to ESRD. Future prospective cohort studies would be helpful to provide more information about the mechanisms relating RDW to DN severity and progression.
